# The effect of calcium hydroxide and double antibiotic paste on radiographic outcomes and periapical MMP-8 levels in regenerative endodontic procedures: a randomized clinical trial

**DOI:** 10.1590/1678-7757-2024-0122

**Published:** 2024-09-20

**Authors:** Burc PEKPINARLI, Mehmet Emin KAVAL, Dilsah COGULU, Betul ILHAN, Timo SORSA, Taina TERVAHARTIALA, Ozant ONCAG

**Affiliations:** 1 Ege University Faculty of Dentistry Department of Pediatric Dentistry Izmir Turkey Ege University, Faculty of Dentistry, Department of Pediatric Dentistry, Izmir, Turkey.; 2 Ege University Faculty of Dentistry Department of Endodontology Izmir Turkey Ege University, Faculty of Dentistry, Department of Endodontology, Izmir, Turkey.; 3 Ege University Faculty of Dentistry Department of Oral and Maxillofacial Radiology Izmir Turkey Ege University, Faculty of Dentistry, Department of Oral and Maxillofacial Radiology, Izmir, Turkey.; 4 University of Helsinki Institute of Dentistry Helsinki University Central Hospital Helsinki Finland University of Helsinki, Institute of Dentistry, Helsinki University Central Hospital (HUCH), Department of Oral and Maxillofacial Diseases, Helsinki, Finland.; 5 Karolinska Institutet Department of Dental Medicine Huddinge Sweden Karolinska Institutet, Department of Dental Medicine, Huddinge, Sweden.

**Keywords:** MMP-8, Radiographic outcomes, Regenerative endodontics, Root canal medicament, Treatment outcomes

## Abstract

In children with poor oral hygiene, dental caries are the most common factor that causes periapical pathologies as a result of irreversible pulpal injuries and leads to the cessation of root development in immature permanent molar teeth.

**Objective:**

The primary goal is to evaluate the effects of two different intracanal medicaments, calcium hydroxide [Ca(OH)_2_] and double antibiotic paste (DAP), on radiographic outcomes during regenerative endodontic procedures (REP) of immature permanent mandibular first molars with symptomatic irreversible pulpitis and symptomatic apical periodontitis (SIP/SAP). Additionally, the secondary goal was to evaluate MMP-8 levels during REP using two different intracanal medicaments.

**Methodology:**

The study included 20 patients with immature mandibular first molars exhibiting SIP/SAP. Participants were randomly assigned into two groups based on the applied intracanal medicament. Ca(OH)_2_ (n=10) was prepared by mixing it with sterile distilled water, while the same amount of powdered metronidazole and ciprofloxacin were mixed and combined with sterile distilled water for DAP (n=10). MMP-8 in periapical samples were measured at baseline and on the 14^th^ day using immunofluorometric assay. Image-J software with TurboReg plug-in was utilized to determine changes in root length, root width, radiographic root area (RRA) during the 12-month follow-up period. Data were analyzed by SPSS 25.0 (*p*<.05).

**Results:**

Significant increase in MMP-8 on the 14^th^ day compared to baseline in both groups (*p*<0.001). There was no significant difference between the two groups in terms of the increase in MMP-8 (*p*>0.05). Root length significantly increased in both groups (*p*=0.001), with Ca(OH)_2_ showing a greater increase (*p*=0.046). Root width and RRA increased similarly in both groups at 12th month.

**Conclusion:**

Both Ca(OH)_2_ and DAP applications resulted in a significant increase in periapical MMP-8 levels. Increase in radiographic root width and root area was similar between two groups, but Ca(OH)_2_ led to a significantly greater increase in root length. Further studies with larger sample sizes are necessary to validate our findings during REP of vital immature permanent mandibular molars. Clinical Trials database: NCT05581706

## Introduction

Deep dental caries in immature mandibular first molars are common in children with poor oral hygiene, often leading to dental pulp and periapical injuries. Symptomatic irreversible pulpitis (SIP) occurs when the dental pulp is irreversibly damaged by caries or pulp exposure, causing sporadic, localized, or radiating pain, which can be spontaneous and worsened by cold stimuli.^1^ Symptomatic apical periodontitis (SAP) is characterized by pain when biting or tapping/pressing the tooth, associated with inflammation in the apical periodontium. The tooth’s response to pulp sensitivity tests may be positive or negative, with radiographs showing periodontal widening and possible apical radiolucency.^1^ In cases where permanent mature molars show radiographic evidence of apical radiolucency alongside clinical symptoms of SIP, treatment decisions focus on effectively controlling bleeding within the remaining pulp tissue after removal of the inflamed segment. Choosing between pulpotomy for vital pulp therapy or pulpectomy for root canal therapy depends on this evaluation.^2-5^ However, managing immature permanent teeth with apical periodontitis is challenging due to thin root canal walls and lack of an apical barrier.^6^ The preferred treatment for such teeth involves continued root development alongside pulp tissue regeneration or repair. Traditional methods such as calcium hydroxide apexifications or MTA obturations often fall short in promoting ongoing root development and leave the tooth prone to fractures.^7,8^ In response, regenerative endodontic procedures (REP) were introduced to facilitate continued root development and apical closure. The primary goal of REP is to alleviate clinical symptoms and heal apical periodontitis, with the secondary goal of increasing root wall thickness and promoting ongoing root formation.^7,8^ This technique fills the pulp space with new, healthy tissue, avoiding the drawbacks of artificial materials such as toxicity and inadequate sealing.^9,10^

In apical periodontitis, the local immune response is highly complex and contains degradation of the extracellular matrix (ECM) components which are the primary constituents of connective tissue.^11,12^ Genetically distinct but structurally related matrix metalloproteinases (MMPs) are an important super-family of zinc and calcium dependent endopeptidases that represent the class of proteolytic enzymes responsible for the degradation and processing of ECM components.^13^ Furthermore, MMPs play a significant role in bone remodeling and resorption in apical periodontitis. In humans, high enzymatic activity for MMPs (MMP-9 and -13) has been reported in periapical granulomas^14^ and the potential role of MMPs (MMP-1, -2, and -3) in pulp tissue destruction of acute, inflamed pulp was shown.^15^ In inflamed tissues, activation of the latent form of MMP-8, which is released by neutrophils, can be triggered by the presence of reactive oxygen metabolites (hydroxyl radicals) and protease mechanisms. These factors may operate independently or cooperatively in the activation process.^16^ Additionally, higher levels of MMP-8 compared to baseline were measured in periapical exudates of necrotic teeth.^15,17^ Due to its known catalytic activities, MMP-8 is believed to play a role in wound healing and tissue remodeling during inflammation. MMP-8 has also been implicated in the pathogenesis of several chronic inflammatory diseases characterized by excessive influx and activation of polymorphonuclear cells (PMNs), including cystic fibrosis, rheumatoid arthritis, periodontal disease, and chronic skin wounds.^18^ However, there is limited data regarding the *in vivo* biological role of MMP-8 in pulpal inflammation and the apical healing process. Two clinical studies on human teeth have indicated that MMP-8 is involved in the formation of intraosseous lesions and that its levels increase in irreversible pulpitis and apical periodontitis in pulpal and periapical tissues.^17,19^ Additionally, two studies using animal models have shown an increase in MMP-8 expression in healthy teeth after root canal treatment with Ca(OH)_2_ medicament.^20^ Furthermore, its role as an anti-inflammatory enzyme during experimental *Porphyromonas gingivalis*-induced periodontitis in mice has been demonstrated.^21^ Despite these findings, the precise role of MMP-8 in inflammation and the healing process remains to be fully elucidated.

To ensure successful healing in REPs, it is crucial to effectively eliminate the infection from the root canal system. Various approaches, such as disinfection and the use of intracanal medicaments, have been reported for this purpose. Many studies advocate using triple antibiotic paste (TAP) (minocycline, metronidazole, and ciprofloxacin) as an intracanal medicament due to its effectiveness against pathogens commonly found in the root canal system.^22^ However, because minocycline in TAP causes dentin discoloration, double antibiotic paste (DAP), which is a mixture of metronidazole and ciprofloxacin, has been proposed as an alternative.^23^ Iwaya, et al.^24^ (2001) demonstrated that REP using DAP in immature permanent teeth with apical periodontitis could lead to increased thickening of dentinal walls, continued root development, and pulp vitality restoration. The use of Ca(OH)_2_ as an intracanal medicament in REPs gained popularity after Chen, et al.^25^ (2012) reported successful cases. Ca(OH)_2_ application has also been proven effective for root canal disinfection during REPs.^26,27^ Additionally, Ca(OH)_2_ intracanal medication has been shown to reduce MMP-8 levels in periapical tissues of necrotic teeth.^17^ Despite numerous *in vivo* studies on the antibacterial effectiveness of Ca(OH)_2_ and antibiotic pastes (TAP/DAP), their effects on periapical inflammatory biomarkers in immature mandibular first molars with SIP and SAP during REPs remain unexplored. With the increasing number of publications and case reports, routine use of REPs for immature molar teeth is encouraged. However, there is still a lack of consensus regarding the choice of medicament during REP. Due to limited data, this study primarily sought to investigate the effects of two different intracanal medicaments on radiographic outcomes during REP of immature permanent mandibular first molars with symptomatic irreversible pulpitis with symptomatic apical periodontitis (SIP/SAP). The secondary goal was defined as the evaluation of periapical MMP-8 levels during REP. The null hypotheses were as follows: *1*) there is no difference between Ca(OH)_2_ and DAP medicament applications regarding the radiographic outcomes during REP, and *2*) the periapical MMP-8 levels during REP are similar when using Ca(OH)_2_ and DAP.

## Methodology

**Figure 1:**
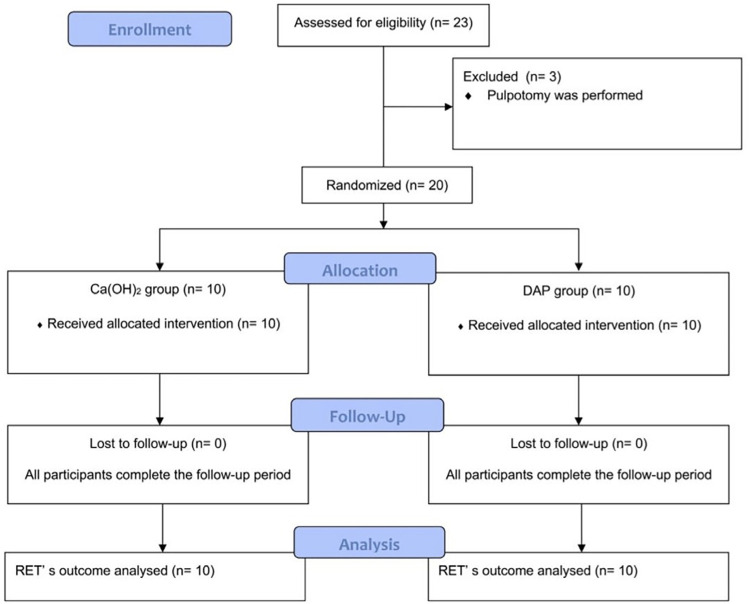
CONSORT 2010-Flow chart.

This randomized clinical trial has been performed in accordance with the Consolidated Standards of Reporting Trials (CONSORT) 2010 guidelines (Figure 1). The study protocol was approved by Research Ethics Committee (17-5.1/10) and was registered in Clinical Trials database (NCT05581706). The sample size was calculated with respect to the primary outcome and was performed using the G*Power 3.1 software (Heinrich Heine University, Dusseldorf, Germany).^28^The calculation (effect size f: 0.40, number of groups: 2), indicated that a sample size of eight teeth per group had an actual power of 80% with a significance level of 0.05. A total of 23 participants were chosen from a pool of patients (aged 6-9 years) who sought treatment at the emergency clinic of the Department of Pediatrics, Faculty of Dentistry, Ege University, between September 2017 and December 2018, due to pain in an immature permanent mandibular first molar. During the initial clinical and radiographic assessment, patients with SIP/SAP were selected and further evaluated. Inclusion criteria were healthy children with vital teeth with SIP/SAP, indication for REP, and informed consent from the patients. We excluded patients with extra oral swelling or luxation with a deep periodontal pocket and for whom application of REP was contraindicated. Additionally, children who were prescribed antibiotics in the previous three months or who were uncooperative during the treatment were excluded from the study. The diagnosis of SIP/SAP was defined as teeth that were particularly sensitive to percussion and palpation, as well as thermal stimuli (especially cold), and had spontaneous, radiating pain that lingered after removal of the stimulus, uncompleted root development and widened/normal periodontal ligament space on initial diagnostic periapical radiographs.^1^ After verbal and written informed consents were obtained from all children and their parents, REPs were administered by a single operator in two sessions, 14 days apart.

### Clinical protocol

The teeth were isolated with a rubber dam (Roeko Dental Dam, Coltene Whaledent, Altstätten, Switzerland) after mandibular anesthesia. Isolated teeth were disinfected with 30% hydrogen peroxide (30 s) and 5.25% sodium hypochlorite (NaOCl) (30 s). Then, the effects of these agents were inactivated with 5% sodium thiosulfate. After meticulous excavation of the carious lesions and attainment of access to the pulp chamber, the removal of the coronal pulp was facilitated through the use of a sterile steel bur and a sharp excavator, ensuring adequate cooling with water irrigation. Following the removal of the coronal pulp tissue, cotton pellets soaked with 0.5% NaOCl were applied for 5 min^3-5^ to confirm the contraindication of vital pulp therapy. The presence of persistent hemorrhage was confirmed visually, and in accordance with the inclusion criteria, teeth that fulfilled the indications for pulpectomy were subjected to REP. Three mandibular molars on which hemostasis was noted underwent pulpotomy and were subsequently excluded from study.

After radiographic determination of working length with an endodontic instrument inserted into the root canals, extirpation was performed using broaches (Mani Inc-Tochigi Ken, Utsunomiyashi, Japan) and inflamed pulp tissue remnants were removed by circumferential filing with a #30 H file (PerfectH Files Stainless Steel: QTY-6, Shenzhen, China), positioned 1 mm behind the radiographic root length with minimal contact with the dentin walls. Removal of pulp tissue was enhanced by performing sodium hypochlorite irrigation, which has strong organic tissue-dissolving activity. Thereafter, root canals were irrigated gently with 20 mL 17% ethylenediaminetetraacetic acid (EDTA), 1.5% NaOCl and sterile distilled water, using a 27-gauge side-vented irrigation needle (C-K Dental Ind. Co., Ltd., Endodontic Irrigation Needles, Seoul, Kore) and dried with sterile paper points. Periapical tissue fluid samples (baseline) were collected by introducing 3 sterile #45 paper points (Meta Biomed Absorbent Paper Points, Cheongju, South Korea) into the distal root canal up to 2 mm through the root apex from the distal canal. After waiting for 1 min, the paper points were withdrawn, the tip was cut from 4 mm, and were transferred to sterile Eppendorf tubes. The samples were stored at 70 °C.^29^

Participants were then randomly assigned into two groups in a 1:1 ratio using an online algorithm (GraphPad Software, Boston, USA). After the randomization procedure, patients were allocated sequential numbers in the order of enrollment. The allocation was managed in a blindfolded fashion by a dental assistant, who delivered the allocated seal to the practitioner just before intracanal medicament placement: Group 1: Calcium hydroxide [Ca(OH)_2_] group, Group 2: Double antibiotic paste (DAP) group. Children and their parents were blinded to applied intracanal medication.

For the Ca(OH)_2_ group, the medicament (Merck, Darmstadt, Germany) was prepared by mixing it with sterile distilled water and then placed in the coronal third of the root canals with a syringe-type carrier. Then, it was tapped down gently with a moist cotton pellet, in line with the previous recommendations and methodologies.^25,26^ For the DAP group, metronidazole (Flagyl, Sanofi-Aventis, Turkiye) and ciprofloxacin (Cipro, Biofarma, Turkiye) powdered antibiotics were stored and sealed in airtight containers. The same proportion of each drug powder (metronidazole and ciprofloxacin) (1:1) was ground into a fine powder to give the paste a cream-like consistency and then mixed with sterile distilled water to form an ointment. DAP was introduced in root canals using a Lentulo spiral filler to fill the entire root canal space 2 mm shorter than the radiographic root canal length. Then, it was tapped down the canal gently with a moist cotton pellet to extend it to the root apex. All medicaments were freshly prepared for each patient. To cover the access cavities, a sterile cotton pellet was placed in the pulp chamber, and the temporary filling was prepared with glass ionomer cement (Ketac Molar Easymix, 3M ESPE, Seefeld, Germany). The patients were informed that they should notify the clinic in case of an unexpected situation and that they should not use painkillers or antibiotics.

**Figure 2:**
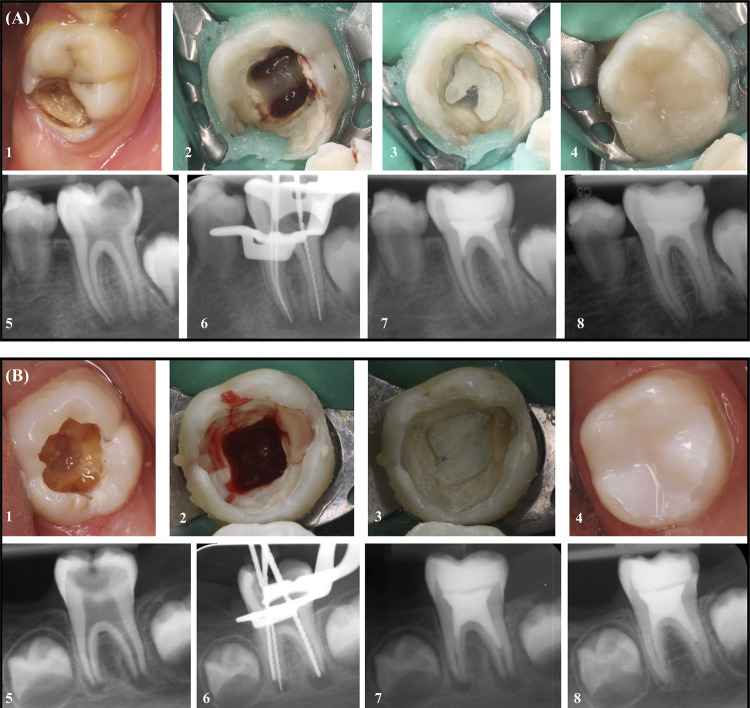
Treatment steps. A: Lower left immature first molar tooth in a 9-year-old boy. 1: Intraoral view before treatment, 2: Bleeding was induced up to CEJ in the root canals, 3: After the bleeding stopped, the entrances of the root canals were closed with 3-4 mm thick MTA, 4: Final composite resin restoration, 5: Initial radiographic image, 6: Determination of working length, 7: Post-op radiographic image 8: 12th month follow up radiographic image. B: Treatment steps. Lower left immature first molar tooth in a 6-year-old girl. 1: Intraoral view before treatment, 2: Bleeding was induced up to CEJ in the root canals, 3: After the bleeding stopped, the entrances of the root canals were closed with 3-4 mm thick MTA, 4: Final composite resin restoration, 5: Initial radiographic image, 6: Determination of working length, 7: Post-op radiographic image 8: 12th month follow up radiographic image.

The patients were recalled after 14 days and the presence of spontaneous pain and tenderness on percussion/palpation was examined. All teeth were asymptomatic at the recall appointment with no pain or tenderness on percussion/palpation and REP was continued. Mandibular anesthesia was performed with lidocaine (Jetokain Simplex, ADEKA, Samsun, Turkey). Rubber dam isolation and asepsis conditions were provided as described in the 1^st^ treatment session. Following the removal of the temporary filling, the medicaments in the root canals were gently removed with 5 mL sterile distilled water irrigation per canal and #30 H file. Periapical tissue fluid samples were obtained from the distal canal using the same protocol as described previously (14^th^ day samples). The root canals were washed with 20 mL of 1.5% NaOCl and 17% EDTA per canal and dried with sterile paper points. Apical bleeding was induced by exiting 2 mm of the root apex with a sterile 2 mm tip of a pre-curved #35 K-file from each canal. The blood was filled up to the cementoenamel junction (CEJ). The openings of the root canals were sealed with 3-4 mm thick MTA (Angelus^®^ MTA White, Londrina, Brazil) after bleeding stopped. MTA was allowed to harden for 15 min, in accordance with the manufacturer’s recommendations. MTA was covered with a resin-modified glass ionomer cement (Shofu Dental Corporation, CX Plus Glass Ionomer Cement, Kyoto, Japan), and the restoration was finished with composite resin (3M Espe Filtek Z250 Hybrid Composite, Seefeld, Germany) (Figure 2). Post-operative periapical radiographs were obtained with the Digora Optime storage phosphor plates (SPP) system (Soredex Corp., Tuusula, Finland) using intraoral film holders to keep the plates parallel to the long axis of the teeth. A size #2 SPP was used for all exposures. SPPs were exposed with a Gendex Oralix DC dental X-ray unit (Gendex Dental Systems, Milan, Italy) operating at 60 kVp, 7 mA, 0.25 s, and the plates were scanned immediately after exposure. The clinical and radiographic follow-up protocol recommended by the European Society of Endodontology (ESE)^30^ was adhered to, and radiographic measurements were conducted on images taken at the 12^th^ month.

### Immunofluorometric assay of MMP-8

Baseline and final MMP-8 concentrations were determined by a time-resolved immunofluorometric assay (IFMA). The monoclonal MMP-8 specific antibodies 8708 and 8706 (Actim Oy, Espoo, Finland) were used as catching antibody and tracer antibody, respectively. The tracer antibody was labeled using europium-chelate.^17^ The assay buffer contained 20 mM Tris-HCl, pH 7.5, 0.5 M NaCl, 5 mM CaCl_**2**_, 50 μM ZnCl_**2**_, 0.5% BSA, 0.05% sodium azide and 20 mg/l diethylenetriaminepentaacetic acid (DTPA). Samples were diluted in assay buffer and incubated for 1 hour, followed by incubation for 1 hour with tracer antibody. The enhancement solution was added and after 5 min and the fluorescence was measured using an EnVision 2105 Multimode Plate Reader (PerkinElmer, Turku, Finland). The detection limit for the assay is 0.08 ng/mL.

### Radiographic evaluation and image analysis

Post-operative and final recall (12^th^ month) digital radiographs were saved and transferred to the Image J software (version 1.47, National Institutes of Health, Bethesda, MD). The standardized radiographs were further aligned using the TurboReg plugin (Biomedical Imaging Group, Swiss Federal Institute of Technology, Lausanne, Switzerland) within the Image J toolkit to minimize any distortions caused by variability in the angulation.^26^ All the images were modified in automatic mode to eliminate any possible investigator bias. An affine distortion algorithm was selected to translate, rotate, shear, skew, enlarge or reduce the source image using the landmarks on the target image. The same three landmarks were selected on both the source and target image. Easily identifiable structures such as mesial CEJ (cementoenamel junction), restoration margins, and apices of adjacent non-erupting mature teeth were selected as landmarks. The aligned target images were saved and used for length and area measurements.

All images were calibrated according to size #2 SPP (vertical dimension 31 mm, horizontal dimension 41 mm) using the “set scale” option in Image J as described by Bose, et al.^26^ (2009). Then, the root lengths, root width, and radiographic root area (RRA) were measured on both preoperative and final recall images to evaluate treatment outcomes. In multirooted teeth, RRA can be difficult to measure when roots overlap.^31,32^ As such, the measurements were performed only in the distal roots due to the presence of superposition in the mesial roots.

**Figure 3:**
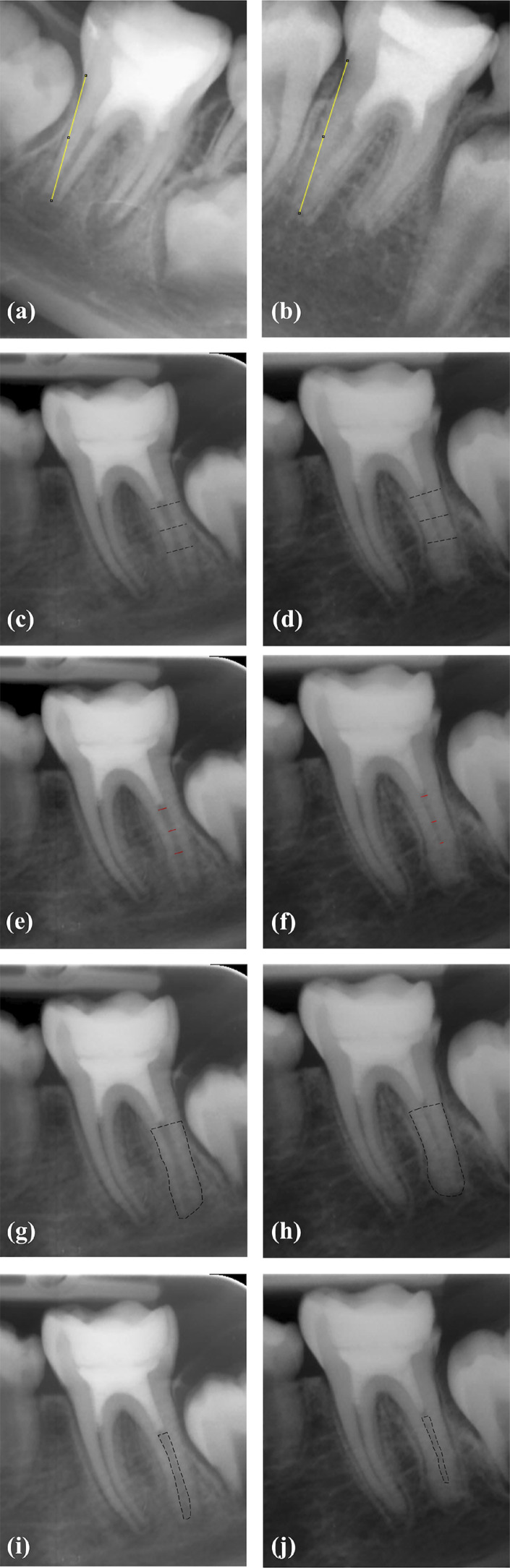
Linear measurements on mathematically aligned radiographic images. Root length: (a) post-operative, (b) 12th month. Root width (c, e) post-operative and (d, f) 12th month (The average root width for preoperative and 12th month images was calculated as; cervical root width-cervical pulp width) + (middle root width -middle pulp width) + (apical root thickness -apical pulp width)/3). RRA (g, i) post-operative and (h, j) 12th month (RRA was defined as the difference between the total root area and the root canal space).

All measurements were performed by the same experienced oral radiologist who was blinded to the study protocol. All the calibrations and measurements were repeated after two weeks to confirm the reproducibility of the procedures. The root length was measured as a straight line from the CEJ to the radiographic apex of the tooth using the “straight-line” tool of Image J.^26^ The root width (dentinal wall thickness) and pulp width for both preoperative and final recall images were measured at the apical, middle, and cervical thirds of each root using the “straight-line” tool. The average root width for both preoperative and final recall images was calculated as: (cervical root width-cervical pulp width) + (middle root width -middle pulp width) + (apical root thickness − apical pulp width)/3.^26^ RRA was defined as the difference between the total root area and the root canal space, and was measured using the polygon selection tool on both post-operative and final recall images. The percentage (%) of increase for root length, average root width, and RRA was calculated as: final recall measurement − post-operative measurement/ post-operative measurement × 100^28^ (Figure 3). We defined the success criteria for radiographic outcomes as the increase in root length, root width, and radiographic root area.

### Statistical analysis

Statistical analysis was performed using IBM SPSS Statistics for Windows, Version 25.0 package program (Released 2017, Armonk, NY: IBM Corp.). Normal distribution of MMP8 data between the two groups was evaluated with the Shapiro-Wilk test using residuals from Analysis of Variance for Repeated Measures. Since non-normal distribution was observed, the difference between the two groups (Ca(OH)_2_ and DAP-independent factor), the difference between the times (baseline and day 14-repeating factor), and group-time interaction were evaluated using the nonparametric Brunner and Langer (model F1-LD-F1) method. Normal distribution of radiological measurements between the two groups was analyzed using the Shapiro-Wilk test. As a normal distribution was observed, the difference between and within the two groups regarding post-operative and final recall measurements as well as the controls for the importance of interaction were analyzed by the analysis of variance (ANOVA) method for repeated measures. In cases in which the interaction was significant, post-operative measurement values and the change in percentile at final recall measurement were compared between the two groups using independent two-group T-test. The differences between post-operative and final-recall measurements within the groups were examined with paired two-sample T-test. Hypothesis checks were performed at a type I error level of 0.05 (*p<*0.05).

## Results

A total of 20 children aged 6 to 9 years (9 females, 11 males) met the inclusion criteria. Three patients were excluded from the study due to clinical indications for vital pulp therapy. The distribution of children’s ages and genders between Ca(OH)_2_ (n=10) and DAP groups (n=10) was statistically similar at baseline (*p*>0.05). No statistically significant difference was observed between the two medicament groups regarding the baseline MMP-8 values (*p*>0.05). There was a statistically significant increase in MMP-8 levels on day 14 compared to baseline in both Ca(OH)_2_ and DAP groups *(p*<0.001). The difference between two groups regarding the increase in MMP-8 levels was not statistically significant (*p*>0.05) (Table 1).

**Table 1:** MMP-8 levels measured at baseline and at the 14th day

Group		Baseline [ng/ml]	Day 14 [ng/ml]
Ca(OH)_2_	N	10	10
	Median	1.0489	3.9683*
	Minimum	0.77	0.81
	Maximum	2.77	83.23
DAP	N	10	10
	Median	0.9524	21.2918*
	Minimum	0.57	2.95
	Maximum	3.49	84.41

*Significant increase in MM8 levels (p < 0.001)

The difference between baseline root lengths (*p*=0.096), root width (*p*=0.734), and RRA (*p*=0.468) for DAP and Ca(OH)_2_ groups was not statistically significant. A statistically significant increase in root length was observed at the 12^th^ month for both the Ca(OH)_2_ (*p<*0.001) and DAP groups (*p*=0.001), with a mean increase of 13.92% (±6.56) for Ca(OH)_2_ and a mean increase by 7.80% (±5.33) for DAP. The mean increase in root length was statistically in favor of Ca(OH)_2_ (*p*=0.046) (Table 2). No significant differences were observed within groups regarding baseline and 12^th^ month measurements for root width and RRA. The mean increase in root width was 15.05% (±7.78) and 12.28% (± 8.69), while the increase in RRA was calculated as 14.52% (±7.14) and 14.61% (±6.32) for Ca(OH)_2_ for DAP respectively. The difference between the two groups regarding 12^th^ month root width (*p*=0.533) and RRA (*p*=0.566) was not significant.

**Table 2:** Change in root length, root width and RRA for Ca(OH)2 and DAP groups at 12th month follow-up

Medicament	N	Increase in root length (%)*	Increase in root width (%)	Increase in radiographic root area (%)
Ca(OH)_2_	10	13.92±6.56	15.05±7.78	14.52±7.14
DAP	10	7.80±5.33	12.28±8.69	14.61±6.32

* The increase in root length was statistically higher in Ca(OH)_2_ group compared to DAP (p = 0.046).

## Discussion

The stage of root development significantly influences the treatment approach for rapidly advancing dentinal caries in immature permanent molars with injured pulp and apical periodontitis. The slender root canals and lack of apical barriers pose challenges in treating these teeth, which complicates matters for both dentists and patients.^8^ An ideal strategy for such cases would seek to facilitate ongoing root development while concurrently regenerating or repairing the pulp tissue. REP has emerged as an alternative to traditional methods in addressing these challenges. Despite the growing number of publications and case reports supporting REP’s efficacy for immature molars, there is still a lack of consensus regarding the choice of medicament during the procedure. Due to limited data, the present study aimed to compare the effects of two different intracanal medicaments with known antimicrobial activity on radiographic outcomes and periapical expression of MMP-8 levels. It is important to emphasize that this study is one of the few studies that conducted REP in vital teeth. In an *in vivo* study using an animal model, Torabinejad, et al.^33^ (2018) investigated the effect of maintaining uninflamed residual apical pulp tissue on the histologic outcome of pulp-dentin complex regeneration after a revascularization procedure in immature ferret cuspid teeth. The authors declared that pulpal amputation a few millimeters short of the apex followed by bleeding allowed the complete regeneration of the normal pulp-dentin complex in immature vital teeth. Recently, this proposed procedure was successfully applied to a vital, inflamed pulp on an immature maxillary central incisor diagnosed with SIP/SAP, which indicates that the use of REP in teeth with symptomatic irreversible pulpitis and open apices has the potential to regenerate a normal pulp-dentin complex.^34^ The present study was conducted on teeth manifesting symptoms consistent with SIP and more importantly SAP, and the inclusion criteria was limited as to include immature mandibular first molars diagnosed with SIP/SAP based on clinical symptoms and radiographic findings.^3^ After the extirpation of the coronal pulp tissue, the presence of persistent hemorrhage was visually verified. In line with the predefined inclusion criteria, teeth meeting the requirements for pulpectomy were subjected to REP. Conventional treatment approaches such as Ca(OH)_2_ apexifications or MTA obturations were not chosen, because these treatment options do not adequately address the underlying issue of root fragility.^6^

Meschi, et al.^35^ (2022) highlighted the insufficient evidence to firmly support regeneration/revitalization for apical periodontitis in (im)mature permanent teeth. However, it is important to highlight that their quantitative analysis included only four studies, predominantly with significant bias issues identified by the authors, such as intervention deviation, randomization and missing data. The authors emphasized the necessity for high-quality clinical trials to enhance the clinical credibility of REPs. In contrast, our study demonstrated noteworthy enhancements in radiological outcomes and periapical MMP-8 levels at the 12-month following REP. While the positive results in treating SIP/SAP are relevant and may address limitations outlined by Meschi, et al.^35^ (2022), they should not be seen as proof of REPs being unequivocally effective. Factors such as patient cooperation, treatment comfort, and long-term tooth survival must be considered when selecting the best approach for managing SIP/SAP in immature mandibular molar teeth in pediatric patients. To the best of our knowledge, this is the first clinical human REP study performed in children with SIP/SAP in immature permanent mandibular first molars. In studies examining the amount of root hard tissue formation in REPs, it has been reported that the blood clot technique causes a significant increase in RRA.^32^ Also, it was stated that a higher percentage of dentinal wall thickening was observed when using antibiotic pastes, while a higher percentage of apical closure was obtained when Ca(OH)_2_ was used in REPs at 12^th^ month follow-ups. However, the increase in root length was similar between antibiotic and Ca(OH)_2_ medicaments.^26^

Ca(OH)_2_ is widely used as an intracanal medicament due to its potent antimicrobial properties. It rapidly releases hydroxyl ions, creating a high pH environment that effectively inhibits nearly all microorganisms in infected root canals after cleaning and shaping procedures.^7,25^ Ca(OH)_2_ also neutralizes endotoxins, aids in mineralization, breaks down organic matter, and forms both a chemical and physical barrier.^7^ Additionally, as a source of hydroxyl radicals, it can lead to the activation of latent pro-MMP-8.^16^ In this study, Ca(OH)_2_ was placed in the coronal part of the root canal, following previous recommendations and methods.^25,26^ Some argue that the high pH from Ca(OH)_2_ might harm essential cells during healing. Conversely, others suggest it could lead to uncontrolled calcification, potentially obstructing odontogenic soft tissue growth. Advocates propose limiting Ca(OH)_2_ to the coronal third of the root canal to harness its benefits while reducing potential toxicity.^25^ DAP on the other hand consists of a mixture of metronidazole and ciprofloxacin and has been shown to be effective in reducing the number of pathogenic microorganisms in infected root canals.^36^

Although antibiotic pastes can be utilized during REPs, the use of Ca(OH)_2_ is recommended, reserving antibiotic pastes for cases involving inflammatory conditions.^9,30^ Furthermore, AAE states that the use of DAP without minocycline paste or substitution of minocycline is a possible alternative as a root canal disinfectant.^37^ Considering the potential adverse effects of minocycline, including discoloration, cytotoxicity, and sensitization, DAP was chosen as the secondary root canal medicament in our study. Nevertheless, caution should be taken regarding the concentration of DAP, as high concentrations may have a negative effect on the survival of SCAPs.^38^ As recommended, DAP was prepared at a concentration of 0.5 mg/mL during clinical procedures.^38^ Fouad, et al.^39^ (2022) reported that the choice of disinfection protocols for use in a randomized controlled trial poses a considerable challenge. On one hand, one must opt for the most effective disinfection methods currently approved for this purpose. On the other hand, it is important to also explore more biocompatible approaches that could potentially foster genuine regeneration of dental pulp and other dental tissues. Fouad, et al.^39^ (2022), investigated the efficacy of three antimicrobial protocols in teeth with necrotic pulp, open apexes and apical periodontitis. The study was conducted on patients undergoing REP, apexification and revascularization, and the endodontic microbiome was analyzed using the 16S ribosomal RNA gene analysis. The results revealed that the reduction in bacterial taxa was significantly lower in the REP group when compared to apexification and revascularization groups. Conversely, the presence of SIP/SAP symptoms in the teeth included in our study suggests their vitality, indicating an earlier stage of inflammation and less bacterial accumulation compared to more advanced, infected necrotic teeth.^40^ Indeed, all teeth included in our study exhibited complete healing without requiring a need for a second medicament dressing session after REP disinfection protocol. This observation could potentially indicate that this procedure is more applicable to vital teeth rather than necrotic ones. However, it also underscores the importance of achieving adequate disinfection of the root canal system while also ensuring the protection of the target cells. Consequently, there may be a delicate balance during the regenerative procedure, allowing for thorough disinfection with minimal harm to cellular structures. Our findings indicate that the use of DAP during REP did not yield any benefits in comparison to Ca(OH)_2_ concerning clinical and radiographic outcomes and the influence on periapical MMP-8. Therefore, it is advisable to consider employing Ca(OH)_2_ in regenerative endodontic procedures due to potential risk of antibiotic-resistant strains associated with the use of antibiotic pastes.^41^

In the present study, previously reported radiographic measurement techniques were applied and increases in root length, root width, and RRA compared to baseline were achieved in immature young permanent teeth after REP.^26,27,32^ When dealing with multirooted teeth, the accuracy of radiographic measurements can be complicated by the overlap of roots.^31^ Consequently, measurements were exclusively conducted in distal roots to avoid the superposition issue in mesial roots. The increase in root length was significantly higher in the Ca(OH)_2_ group when compared to the DAP medicament group. Therefore, the primary null hypothesis was partially rejected.

The MMP-8 levels were evaluated to determine the proinflammatory changes in the periapical area due to lack of data regarding its role during the progression and assessment of apical periodontitis in human teeth with immature root development.^42^ To establish a connection between periapical MMP-8 values and radiographic measurements, the sampling process was also limited to the distal canal in the present study. Additionally, opting for the distal root canal during sampling yields a larger amount of tissue fluid in a single procedure.^43^ This choice is supported by the fact that the distal canal of mandibular first molars is notably wider. It has been reported that MMP-8 plays a role in the formation of intraosseous lesions, and its level increase in irreversible pulpitis and apical periodontitis in pulpal and periapical tissues.^17,19^ Wahlgren, et al.^17^ (2002) observed a decrease in MMP-8 levels following root canal treatment using Ca(OH)_2_ in teeth with necrotic pulp and periapical granulomas. This decrease was linked to a decline in PMNs, known contributors to inflammation during the healing process. In contrast to Wahlgren, et al.^17^ (2002) our study focused on vital teeth without apical granuloma, as confirmed through clinical and radiographic assessments, revealing an increase in MMP-8 levels compared to baseline in both Ca(OH)_2_ and DAP groups during REP. The discrepancies between the two studies could be attributed to differences in tooth selection criteria, root development stage, and the extent of pulpal/periapical inflammation in the included teeth. Additionally, the increased MMP-8 levels might stem from mesenchymal cells like fibroblasts and odontoblasts in the surviving apical papilla and Hertwig’s epithelial root sheath (HERS),^20,21^ despite the presence of irreversible pulpal inflammation in immature teeth. Nevertheless, similar to our findings, an increase in MMP-8 expression was also observed in healthy teeth after root canal treatment in two sessions using Ca(OH)_2_ medicament in an animal study.^20^ It was shown that MMP-8 acts as an anti-inflammatory enzyme during acute lung injury and experimental *Porphyromonas gingivalis* induced periodontitis in mice.^20,21^ The authors suggested that the anti-inflammatory role of MMP8 may be due to the breakdown and elimination of components that participate in the increase of inflammation as well as to process and activate anti-inflammatory mediators.^18,21^ The anti-inflammatory properties of MMP-8 were also associated with the inhibition of the release of proteolytic enzymes from PMN cells (elastase, cathepsin G, MMP-8).^44^ However, the role of MMP-8, which has been reported as an inflammatory enzyme in apical periodontitis, is still not fully understood and further studies are needed to clarify its potential influence.^13^

In teeth with incomplete root development, the apical papilla is a reservoir of mesenchymal stem cells (SCAP).^44^ During regenerative endodontic procedures, induced bleeding helps in the migration of SCAPs into the root canal.^9^ SCAPs survive in low-oxygen environments, supported by the vascularized periradicular granulomas typical in apical periodontitis.^45^ Hertwig’s epithelial root sheath (HERS), present even in apical periodontitis and abscesses, may contribute to root development and healing alongside SCAPs.^9,46^ Our research found that elevated periapical MMP-8 levels were correlated with the healing process. This might be due to MMP-8 production from mesenchymal stem cell sources, including SCAPs and HERS, which are resilient even in apical periodontitis.^47^ The increase in MMP-8 levels after REP may be related to mesenchymal cell activity in the periapical region, contributing to the enzyme’s anti-inflammatory properties.^16,20,22^ Despite evidence of proMMP-8 activation by hydroxyl ions from calcium hydroxide,^16^ our study did not find a statistically significant difference between Ca(OH)_2_ and DAP regarding MMP-8 level increases.

The primary limitation of this study is the small sample size of treated teeth. In clinical trials involving children aged 6 to 10 years, clinicians face substantial challenges related to child cooperation, measurement of pain (due to its subjectivity especially in children), isolation difficulties, acceptance of local anesthesia, appointment compliance, and the application of rubber dams, which all can restrict the sample size.^28,48,49^ Furthermore, the application of REP, a more challenging, but more advantageous procedure compared to traditional apexification, may have contributed to the limitation in sample size. Studies encompassing larger sample sizes are still necessary to obtain a more thorough insight into the effectiveness of REPs in addressing immature mandibular molars with SIP/SAP. Nevertheless, as one of the few studies that have undertaken REP in vital teeth and the pioneering clinical human REP study conducted in children with SIP/SAP affecting immature permanent mandibular first molars, our findings demonstrated the potential effectiveness of REP as an alternative treatment approach to MTA apexification in vital immature permanent mandibular first molars that warrant pulpectomy due to apical periodontitis. Furthermore, the potential involvement of MMP8 suggests that this enzyme, known for its inflammatory role in irreversible pulp and periapical inflammation, might also participate in the periapical healing process of vital, immature mandibular first molars. This may be true even in the presence of apical periodontitis, possibly due to the existence of mesenchymal stem cell sources like SCAP and HERS that can endure under such conditions. Our results also highlight that using DAP during REP did not grant any advantages over Ca(OH)_2_ in terms of clinical and radiographic outcomes and its impact on periapical MMP-8. Consequently, it is advisable to consider employing Ca(OH)_2_ in regenerative endodontic procedures due to potential risks associated with antibiotic pastes. Our findings are promising, as they provide insight and support for the utilization of REPs in treating immature permanent mandibular first molars with SIP/SAP.

## Conclusion

This study evaluated the effects of Ca(OH)_2_ and DAP on radiographic outcomes and periapical MMP-8 levels during REPs of immature permanent mandibular first molars with SIP/SAP. No significant differences was observed between two medicaments regarding the MMP-8 levels on day 14^th^, and radiographic root width and root area at 12^th^ month. However, the increase in radiographic root length was statistically higher in Ca(OH)_2_ group compared to DAP. Further studies with larger sample sizes and long-term follow-up over 12 months are required to confirm our findings and clarify the influence of Ca(OH)_2_ and DAP medicaments on radiographic outcomes and periapical MMP-8 levels during REPs of human vital permanent teeth with immature root development.
